# Lack of MOF Decreases Susceptibility to Hypoxia and Promotes Multidrug Resistance in Hepatocellular Carcinoma via HIF-1α

**DOI:** 10.3389/fcell.2021.718707

**Published:** 2021-09-01

**Authors:** Meng Wang, Haoyu Liu, Xu Zhang, Wenbo Zhao, Xiaoyan Lin, Fei Zhang, Danyang Li, Chengpeng Xu, Fei Xie, Zhen Wu, Qibing Yang, Xiangzhi Li

**Affiliations:** ^1^Shandong Provincial Key Laboratory of Animal Cell and Developmental Biology, School of Life Sciences, Advanced Medical Research Institute, Shandong University, Qingdao, China; ^2^Department of Cell and Neurobiology, School of Basic Medical Sciences, Shandong University, Jinan, China; ^3^Department of Hematology, Shandong Provincial Hospital Affiliated to Shandong First Medical University, Jinan, China; ^4^Department of Pathology, Shandong Provincial Hospital Affiliated to Shandong First Medical University, Jinan, China; ^5^Department of Rehabilitation, Qilu Hospital of Shandong University, Jinan, China

**Keywords:** MOF, hepatocellular carcinoma, hypoxia inducible factor-1α, hypoxia, protein acetylation, drug resistance

## Abstract

Hypoxia-inducible factor-1α (HIF-1α) promotes oncogenesis in hepatocellular carcinoma and is functionally linked to cell proliferation, chemoresistance, metastasis and angiogenesis. It has been confirmed that the low expression level of Males absent on the first (MOF) in hepatocellular carcinoma leads to poor prognosis of patients. However, potential regulatory mechanisms of MOF in response to hypoxia remain elusive. Our results demonstrate that MOF expression is negatively associated with HIF-1α expression in hepatocellular carcinoma tissues and in response to chloride-mimicked hypoxia in hepatocellular carcinoma cell lines. MOF regulates HIF-1α mRNA expression and also directly binds to HIF-1α to mediate HIF-1α N-terminal lysine acetylation, ubiquitination and degradation, with downstream effects on MDR1 levels. Functional inactivation of MOF enhances HIF-1α stability and causes cell tolerance to hypoxia that is insensitive to histone deacetylase inhibitor treatment. Dysfunction of MOF in hepatocellular carcinoma cells also results in chemoresistance to trichostatin A, sorafenib and 5-fluorouracil via HIF-1α. Our results suggest that MOF regulates hypoxia tolerance and drug resistance in hepatocellular carcinoma cells by modulating both HIF-1α mRNA expression and N-terminal acetylation of HIF-1α, providing molecular insight into MOF-dependent oncogenic function of hepatocellular carcinoma cells.

## Introduction

Liver cancer is the fourth most diagnosed cancer type and the third leading cause of cancer-related death in China (Feng et al., 2019). Worldwide, the incidence of liver cancer ranks sixth and the fatality rate ranks fourth (Bray et al., 2018). Hepatocellular carcinoma (HCC) accounts for the majority of liver cancer, reaching 75–85% of cases (Bray et al., 2018). Despite focused efforts to develop effective treatments for HCC, the 5-year survival rate of patients with HCC remains low, in part attributed to tumor chemoresistance (Su et al., 2018).

In human tumors, intratumor regions away from blood vessels are often hypoxic (Wilson and Hay, 2011). The orthotopic HCC models have observed a significant decrease in the partial pressure of oxygen within the tumor (Cater et al., 1963; Riedl et al., 2008; Liu et al., 2014; Wang et al., 2017). At the same time, oxygen probe measurements in the orthotopic HCC rat model show severe hypoxia in HCC (Riedl et al., 2008). Hypoxia marker is considered to be an independent poor prognostic factor for HCC (Xiong et al., 2017). Hypoxia-inducible factor-1 alpha (HIF-1α) is a master regulator of cellular and developmental responses to hypoxia (Lee et al., 2020). Several studies found that about 60% of HCC samples had positive HIF-1α expression, accompanied by a significant poor prognosis (Srivastava et al., 2015; El Shorbagy et al., 2020). By regulating its downstream multidrug resistance protein 1 (MDR1)/P-glycoprotein 1 gene, HIF-1α has been identified as a multidrug resistance regulator in a variety of tumors, with a reported role in mediating chemoresistance to 5-fluorouracil (5-FU), gemcitabine, oxaliplatin, and sorafenib (Liu et al., 2008; Liang et al., 2013; Chen J. et al., 2014; Wu et al., 2016; Schoning et al., 2017; Shukla et al., 2017; Qin et al., 2018; Xu et al., 2019). In HCC, HIF-1α functions as an oncogene, mediating cell proliferation, tumor chemoresistance, metastasis, glycolysis and angiogenesis (Wu et al., 2016; Abu-Remaileh et al., 2018; Qin et al., 2018; Wen et al., 2019). HIF-1α is regulated at both the transcriptional and posttranslational levels. Deacetylation at N-terminal lysine residues (K10, K11, K12, K19, and K21) (Geng et al., 2011; Zhang et al., 2017) and acetylation at C-terminal lysine residues (K532, K674, and K709) (Xenaki et al., 2008; Lim et al., 2010; Geng et al., 2012) have been shown to enhance the protein stability of HIF-1α, thus promoting tumor cell tolerance to hypoxic conditions (Geng et al., 2011). HIF-1α acetylation is regulated by lysine acetyltransferases (KATs) and histone deacetylases (HDACs). The protein stability of HIF-1α is enhanced by HDAC4 through deacetylation of N-terminal lysine residues of HIF-1α (Geng et al., 2011). Accordingly, treatment of cells with HDAC inhibitors, such as trichostatin A (TSA), leads to HIF-1α degradation (Schoepflin et al., 2016).

Males absent on the first (MOF) is originally discovered in *Drosophila*, also known as KAT8 or MYST1, that specifically acetylates lysine 16 of histone H4 and many non-histone proteins such as p53 and NRF2 (Li et al., 2009, 2010, 2012; Chen Z. et al., 2014). MOF plays important roles in many physiological processes, such as cell stemness, cell cycle, and embryonic development (Li et al., 2009, 2010, 2012; Fullgrabe et al., 2013; Jaganathan et al., 2014; Tawana et al., 2015). Furthermore, recent studies have also indicated an emerging role of MOF in tumorigenesis (Wang et al., 2013; Chen Z. et al., 2014; Jaganathan et al., 2014). It has been investigated that low expression of MOF leads to enhanced HCC cell proliferation and poor prognosis of patients (Zhang et al., 2014). However, in process of HCC hypoxia, the expression pattern and function of MOF have not been well investigated. Here, we demonstrate for the first time that low level of MOF promotes HIF-1α transcription and N-terminal lysine acetylation, and simultaneously promotes tolerance of HCC cells to hypoxia and chemoresistance. These results provide a novel mechanism for chemoresistance that may be useful in the development of targeted approaches to treat HCC.

## Materials and Methods

### Antibodies and Reagents

Antibody against MOF (A300-992A) was purchased from Bethyl Laboratories (Montgomery, TX, United States) and used for western blot and IHC experiments. MOF (sc-81765) antibody was used for co-immunoprecipitation (CoIP), immunofluorescence and chromatin immunoprecipitation (ChIP) was purchased from Santa Cruz Biotechnology (Dallas, TX, United States). HIF-1α (BF0593, Affinity Biosciences, Cincinnati, OH, United States) was used for IHC experiments. 6 × His Tag (66005-1-Ig), HIF-1α (20960-1-AP, used for both western blot and immunofluorescence), Myc-Tag (60003-2-Ig), Flag-Tag (66008-3-Ig), HA-Tag (66006-2-Ig) and β-Actin (60009-1-Ig) were purchased from Proteintech (Wuhan, China). Cleaved Caspase-3 (AF7022) was purchased from Affinity Biosciences. Pan-lysine acetylation (PTM-102) antibodies were purchased from PTM Bio (Hangzhou, China). Rabbit Control IgG (AC005) and Mouse Control IgG (AC011) were purchased from ABclonal (Wuhan, China). The secondary HRP-conjugated goat anti-rabbit (111-035-003) and goat anti-mouse (115-035-003) antibodies, Cy3-conjugated goat anti-mouse (115-165-003) and Alexa Fluor 488-conjugated goat anti-rabbit (111-545-003) were obtained from Jackson ImmunoResearch (West Grove, PA, United States). MOF inhibitor MG149, eukaryote protein synthesis inhibitor CHX, proteasome inhibitor MG132, HDAC inhibitor TSA, and HIF-1α inhibitor LW6 were purchased from MedChemExpress (MCE, Princeton, NJ, United States). 5-FU, CoCl_2_, MTT and Tween 20 were purchased from Aladdin (Shanghai, China). Sorafenib was purchased from Meilun Biotechnology (Dalian, China). CHX, MG149, MG132, TSA, LW6, 5-FU, and sorafenib were dissolved in DMSO. CoCl_2_ was dissolved in sterilized ultrapure water. MTT was dissolved in phosphate-buffered saline (PBS).

### Patients and Specimens

A total of 86 HCC patients at the Shandong Provincial Hospital Affiliated to Shandong University (Jinan, China) were enrolled in this study. Formalin-fixed, paraffin embedded tumor tissues of 48 patients were processed into sections for subsequent IHC experiments. In addition, 38 pairs of HCC tissues and corresponding peritumoral tissues were collected during surgery and immediately placed in liquid nitrogen for further protein extraction and western blot assay. All subjects provided informed consent for inclusion before they participated in the study. The study was conducted in accordance with the 1975 Declaration of Helsinki, revised in 2013. The protocol was approved by the Ethics Committee of Shandong Provincial Hospital Affiliated to Shandong University (NSFC: NO. 2018-074).

### IHC Staining and Evaluation

Following deparaffinization and quenching of endogenous peroxidase, sections were incubated with 10% fetal bovine serum (FBS) (Gibco, Waltham, MA, United States) in PBS. Subsequently, slides were incubated with HIF-1α (1:200) and MOF (1:200) antibodies dissolved in PBS for 4°C overnight. Sections were then incubated with secondary antibody and get stained by DAB Detection Kit (Polymer) (Gene Tech, Shanghai, China) according to the protocol. Slides were counterstained with Hematoxylin (Solarbio, Beijing, China) and observed under a microscope (Nikon, Tokyo, Japan). IHC staining was conducted based on staining intensity of positive-staining cells. The staining intensity or positive cell percentage was divided into four degrees and scores (For staining intensity, 3, strong; 2, moderate; 1, weak; 0, negative; and for positive cells, 3, >75%; 2, 50–75%; 1, 25–50%; 0, <25%). The final IHC score was decided by adding intensity degree and percentage score.

### Cell Lines and Transfection

Huh-7 and Hep3B cells were purchased from Procell (Wuhan, China) and cultured in RPMI-1640 (M&C, Beijing, China) containing 10% FBS (LONZA, Shanghai, China) at 37°C with 5% CO_2_ in a humidified chamber. Plasmid transfections were performed with JetPRIME (Polyplus, Strasbourg, France) according to the manufacturer’s protocol.

### Plasmid Construction

Two different MOF shRNA (shMOF #1 and shMOF #2) sequences were inserted into pGPU6/GFP/Neo, as well as a blank vector (shVector) were purchased from GenePharma (Shanghai, China). Human Flag/6 × His-MOF overexpression plasmid and a blank vector (Vector) was purchased from Vigenebio (Jinan, China), and mutant MOF plasmid with Flag-Tag (Flag-MOF-mu) was used as previously described (Li et al., 2010). HA-Ub plasmid was obtained from Addgene (Watertown, MA, United States). To generate Myc-HIF-1α-wt plasmid, human HIF-1α sequence was amplified from cDNA library of Huh-7 cells and cloned into pcDNA3-myc vector at restriction sites of *Bam*HI and *Xba*I (TOYOBO, Osaka, Japan). Hieff Mut^TM^ Multi Site-Directed Mutagenesis Kit (Yeasen, Shanghai, China) was used to generate mutant HIF-1α (Myc-HIF-1α-mu) plasmid that contained lysine-to-arginine mutation at residues K10, K11, K12, K19, and K21. We used Plasmid-F and Site #1-3-R, and Myc-HIF-1α-wt as template to generate fragment 1, Site #1-3-F and Plasmid-R were used to generate fragment 2. Then we used Ex-F and Plasmid-R to generate fragment 3 as the template was fragment 2. Using fragment 1 and 3 as templates, Plasmid-F and -R as primers, we generated HIF-1α sequence with three mutations. We ligated mutated HIF-1α sequence to pcDNA3-myc vector at restriction sites of *Bam*HI and *Xba*I. Next, we used Site #4 and Site #5 primers to mutate this plasmid and generated Myc-HIF-1α-mu plasmid with five complete mutations according to the manufacturer’s protocol. The shRNA and primer sequences used for vector construction were listed in Supplementary Table 1.

### RNA Extraction, Reverse Transcription, and Quantitative Real Time PCR (qRT-PCR)

Total RNA was extracted with RNAiso Plus (Takara, Kyoto, Japan). Complementary DNA was generated using RevertAid First Strand cDNA Synthesis Kit (Thermo Fisher Scientific, Waltham, MA, United States) according to the protocol. qRT-PCR was performed on a LineGene 4840 Real-time PCR system (Bioer, Hangzhou, China) using THUNDERBIRD SYBR qPCR Mix (TOYOBO). Quantitation of the relative expression levels of each gene were performed in triplicate and calculated using 2^–ΔΔCT^ method. β-Actin was used as an endogenous control. The primer sequences for detecting genes were listed in Supplementary Table 2.

### Western Blot Assay

Total protein was extracted by sodium dodecyl sulfate lysis buffer (1% sodium dodecyl sulfate, 5% glycerol, 1 mM ethylene diamine tetraacetic acid (EDTA), 25 mM Tris, and 150 mM NaCl) with phenylmethylsulfonyl fluoride. Protein samples were separated by 10% sodium dodecyl sulfate–polyacrylamide gel electrophoresis and were transferred to polyvinylidene fluoride membrane (Millipore, Bedford, MA, United States). After blocking with 5% non-fat milk at room temperature for 1 h, membranes were probed with primary antibodies at 4°C overnight. Membranes were then washed using Tris-buffered saline with Tween 20 (TBST) for four times and incubated with corresponding secondary antibodies at room temperature for 1 h. Bound antibodies were visualized using an enhanced chemiluminescence kit (Wanleibio, Dalian, China). Primary antibodies were dissolved in TBST with 3% bovine serum albumin (1:1000). Secondary antibodies were dissolved in TBST (1:5000).

### Immunofluorescence Assays

2 × 10^4^ Cells were seeded in a 24-well plate containing cell culture slides and cultured overnight. Then the medium was replaced with a medium containing 100 μM CoCl_2_ and cultured for 24 h. Fixed the cells with 1% formaldehyde for 15 min at room temperature, washed using PBS to remove the formaldehyde, used PBS containing 5‰ Tween-20 to incubate the slide for 20 min at room temperature. The slides were incubated for 1 h at room temperature with 10% FBS. The primary antibodies (1:200) were incubated overnight at 4°C, and the secondary antibodies (1:500) were incubated at room temperature for 2 h. DAPI (C0060, Solarbio) was used to indicate the nucleus. The DP74 color fluorescence camera (Olympus, Tokyo, Japan) was used to observe cell immunofluorescence.

### Nuclear and Cytoplasmic Protein Extraction

Nuclear and cytoplasmic protein extraction assay was performed with Nuclear and Cytoplasmic Protein Extraction Kit (P0027, Beyotime, Beijing, China) according to the manufacturer’s protocol.

### CoIP, Polyubiquitination, and Protein Acetylation Assays

Cells were lysed in BC-200 cell lysis buffer (20 mM HEPES buffer, pH 7.9, containing 200 mM KCl, 1 mM EDTA, 10 mM β-mercaptoethanol, 0.1% NP-40 and 10% glycerol) with protease inhibitor cocktail (APExBIO, Houston, TX, United States). CoIP assay was performed using Protein A/G magnetic beads (Bimake, Shanghai, China) according to the manufacturer’s protocol. 1 μg of antibody was added for each CoIP assay. Wash buffer in the protocol was replaced by BC-200 cell lysis buffer with protease inhibitor cocktail. For polyubiquitination and acetylation assay, cells were pretreated with 100 μM CoCl_2_ and 10 μM MG132 for 24 h prior to CoIP assay. After the protein was obtained by CoIP, the acetylation of HIF-1α was detected using pan-lysine acetylation antibody (Zhang et al., 2017).

### Cytotoxicity Assay

For cytotoxic assays, 2,500 cells were seeded in 96-well plates and cultured overnight. Before treatment, culture medium was replaced with fresh medium containing reagents. Cells were then incubated for 72 h. Four hours before the end of incubation, MTT solution (5 mg/ml, 10 μl) was added to each well. The culture medium was then removed and DMSO (100 μl) was added to dissolve formazan crystals. The absorbance value was determined at 450 nm using a SPECTROstar Nano (BMG LABTECH GmbH, Ortenberg, German). Three parallel experiments were performed for each gradient.

### Cell Viability Assay

Cells were seeded at 2500 per well into 96-well culture plates and incubated for 0, 24, 48, 72, 96, or 120 h. After incubation, 10 μl of MTT was added to each well and the cells were incubated for 4 h according to the manufacturer’s instructions. Absorbance values were determined at 450 nm using SPECTROstar Nano. Three parallel experiments were performed for each time point.

### ChIP Assay

ChIP assay was performed according to the Upstate Biotechnology ChIP protocol (Upstate Biotechnology, Lake Placid, NY, United States). The immunoprecipitated DNA was quantified by qRT-PCR. The primer sequences are listed in Supplementary Table 2.

### Luciferase Assay

Luciferase assay was performed as previously described (Wang et al., 2021). Huh-7 cells and Flag/6 × His-MOF plasmid were used in this experiment. The primers used to construct luciferase plasmid are listed in Supplementary Table 1.

### Statistical Analysis

All statistical data are presented as means ± standard deviation. All data were analyzed using GraphPad Prism software (San Diego, CA, United States). Chi-square tests were used to analyze pathological data. Cell viability and cytotoxicity results were assessed by two-way ANOVA. The Student’s *t*-test was applied to qRT-PCR data. A value of *P* < 0.05 was considered statistically significant.

## Results

### MOF Expression Is Negatively Correlated With HIF-1α in HCC Tissues and Under Cell Hypoxia

**FIGURE 1:**
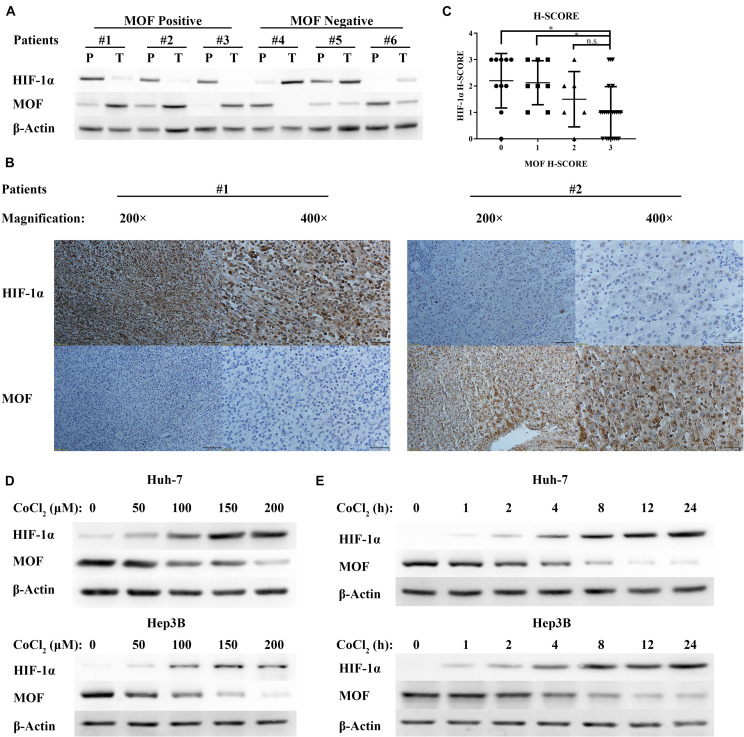
MOF expression is negatively correlated with HIF-1α expression in HCC tissues and in response to hypoxia. **(A)** Representative western blots of MOF and HIF-1α in HCC tissues (T) and their paired peritumoral tissues (P). **(B)** IHC staining images of representative HCC tissues with antibodies against human MOF and HIF-1α, and **(C)** statistical relationship between MOF and HIF-1α scores in HCC. Chi-square values: 0–3, 10.49, df = 3; 1–3, 8.574, df = 3; 2–3, 4.875, df = 3. Scale bar: 100 μm (left in #1 and #2), 50 μm (right in #1 and #2). **(D,E)** Western blots of MOF and HIF-1α in Huh-7 (upper panel) and Hep3B (lower panel) cells upon exposure to increasing concentrations of CoCl_2_-mimicked hypoxia for 24 h **(D)**, or to 100 μM CoCl_2_ over a time course **(E)**. ^∗^*P* < 0.05; n.s., not significant.

Males absent on the first expression has been shown to be down-regulated in HCC (Zhang et al., 2014). However, there is no research evaluating the relationship between MOF expression and HIF-1α expression in liver cancer. In order to determine the correlation between MOF and HIF-1α, we performed western blot assays for 38 pairs of human HCC tumors (T) and peritumoral tissues (P). MOF and HIF-1α expression showed a consistent pattern of negative correlation (Figure 1A). Samples with low expression of MOF in tumor tissue relative to peritumoral tissue had significantly higher HIF-1α expression, and vice versa (Table 1). To verify these results, we also detected MOF and HIF-1α expression levels in 48 human HCC tissues by immunohistochemistry (IHC) staining (Figure 1B). The MOF and HIF-1α staining scores were significantly negatively correlated (Figure 1C). When the MOF score is low (H-Score = 0 or 1), the HIF-1α score is higher; but when the MOF score is high (H-Score = 3), the score of HIF-1α is lower.

**TABLE 1:** Western blot analysis of MOF and HIF-1α in HCC tissues.

	HIF-1α positive	HIF-1α negative	Total
MOF positive	6	15	21
MOF negative	14	3	17
Total	20	18	38

*Two-sided Pearson Chi-Square tests: Value = 10.90, df = 1, *P* = 0.0010, OR = 0.3469, 95% CI = 0.1637 to 0.6598.*

Cobalt chloride (CoCl_2_) treatment has been widely used for mimetic hypoxic conditions. Thus, to identify the relationship between MOF and HIF-1α expression under hypoxic condition, we treated Huh-7 and Hep3B HCC cells with CoCl_2_. With increasing CoCl_2_ concentration (Figure 1D) or dose (Figure 1E), the protein levels of HIF-1α were upregulated, while the protein levels of MOF were decreased. Therefore, hypoxic conditions have opposite effects on MOF and HIF-1α expression.

### HIF-1α Expression Is Regulated in a MOF-Dependent Manner

**FIGURE 2:**
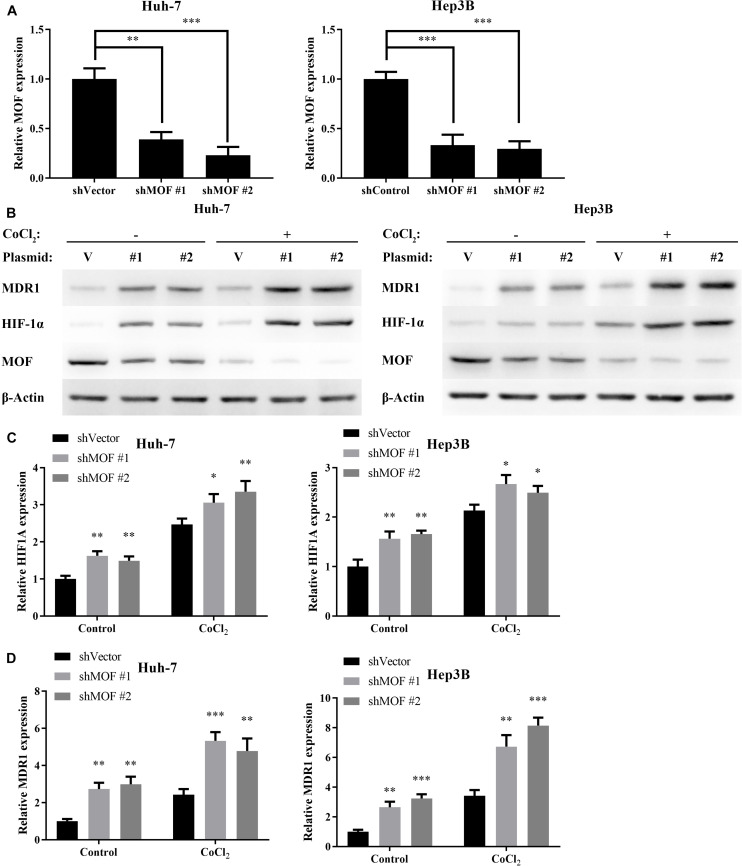
MOF knockdown increases HIF-1α and MDR1 expression. **(A)** qRT-PCR showed decreased MOF mRNA levels in Huh-7 and Hep3B cells transfected with two different MOF shRNA plasmids or a control plasmid. **(B)** qRT-PCR of HIF-1α mRNA expression in cells with control (shVector) or MOF knockdown (shMOF #1 or #2) under normoxic or CoCl_2_-induced hypoxic conditions. **(C)** qRT-PCR of MDR1 mRNA expression in cells with control or MOF knockdown under normoxic or hypoxic conditions. **(D)** Representative western blots of MOF, HIF-1α and MDR1 in control or shMOF knockdown cells under normal or hypoxic conditions. Cells were treated with 100 μM CoCl_2_ or equal volume of PBS for 24 h. V, shVector; #1, shMOF #1; #2, shMOF #2. ^∗^*P* < 0.05, ^∗∗^*P* < 0.01, and ***P < 0.001 as compared to shVector.

**FIGURE 3:**
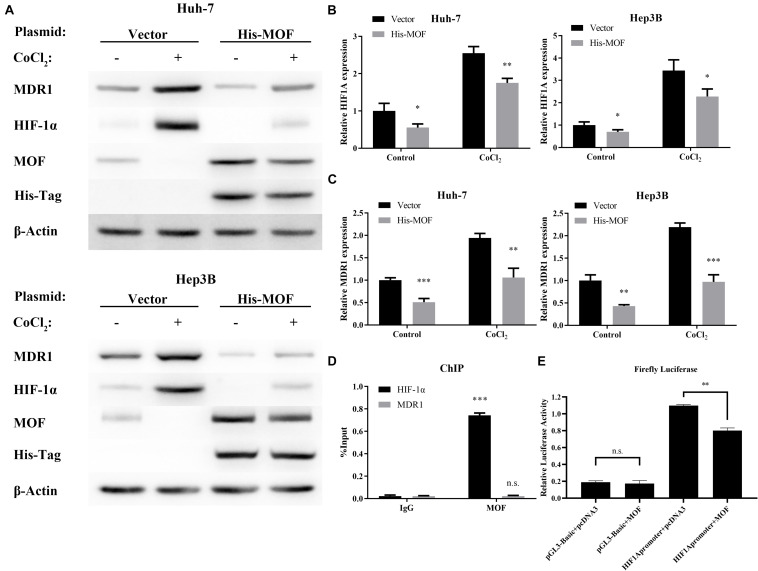
MOF overexpression decreases HIF-1α and MDR1 expression. **(A)** qRT-PCR showed mRNA expression levels of HIF-1α in Huh-7 and Hep3B cells transfected with MOF overexpression vector compared with a blank vector. **(B)** qRT-PCR showed mRNA expression levels of MDR1 in Huh-7 and Hep3B cells transfected with MOF overexpression vector compared with a blank vector. **(C)** Western blot assay of MOF, HIF-1α, MDR1 and His-Tag in cells transfected with a MOF overexpression vector compared with a blank vector transfection. **(D)** ChIP assay showed MOF binding at the HIF-1α versus MDR1 gene locus. **(E)** Detection of luciferase activity of the HIF-1α promoter site in Huh-7 cell line. Cells were treated with 100 μM CoCl_2_ or equal volume of PBS for 24 h. ^∗^*P* < 0.05, ^∗∗^*P* < 0.01, and ^∗∗∗^*P* < 0.001 as compared to Vector; n.s., not significant.

To investigate whether MOF may be involved in regulating HIF-1α expression, we used two different shRNA vectors for transient transfection into Huh-7 and Hep3B cell lines, with each shRNA resulting in MOF knockdown (Figure 2A). HIF-1α mRNA levels were significantly upregulated after knockdown of MOF, both under normoxic and hypoxic conditions (Figure 2B). Furthermore, MDR1, which is known to be transcriptionally regulated downstream of HIF-1α (Qin et al., 2018), showed a similar pattern of increased mRNA expression in shMOF cells (Figure 2C). Similar results were observed at the protein level (Figure 2D). It has been confirmed that HIF-1α mainly stimulates transcription of its target genes in the nucleus (Dengler et al., 2014). So, we conducted nuclear and cytoplasmic protein separation experiments (Supplementary Figure 1A). Clear evidence showed that the lack of MOF led to significant upregulation of HIF-1α in both nucleus and cytoplasm. Conversely, transfection of a MOF overexpression vector significantly reduced the mRNA levels of HIF-1α (Figure 3A) and MDR1 (Figure 3B), with consistent results observed at the protein level (Figure 3C).

As a transcriptional regulator, the binding of MOF at gene locus may lead to alteration in gene expression level (Li et al., 2012). We used the previous ChIP-Seq data and HOMER (Heinz et al., 2010; Li et al., 2012) to analyze the MOF binding motif and the binding site, and found that MOF has a binding region at the promoter of HIF-1α (Supplementary Table 3). Therefore, we performed Chromatin immunoprecipitation (ChIP) experiments to evaluate whether MOF may directly bind to the HIF-1α and MDR1 gene locus. The results suggest that MOF binds to the HIF-1α gene locus, while no MOF binding was detected at the MDR1 gene locus (Figure 3D). We also performed the luciferase assay to verify and found that overexpression of MOF in cells can reduce the expression level of HIF-1α (Figure 3E). This indicates that MOF directly regulates HIF-1α at the transcriptional level by binding to the HIF-1α gene locus, whereas regulation of MDR1 expression by MOF is likely to occur indirectly through HIF-1α.

### MOF Interacts With HIF-1α and Mediates Its N-Terminal Acetylation

**FIGURE 4:**
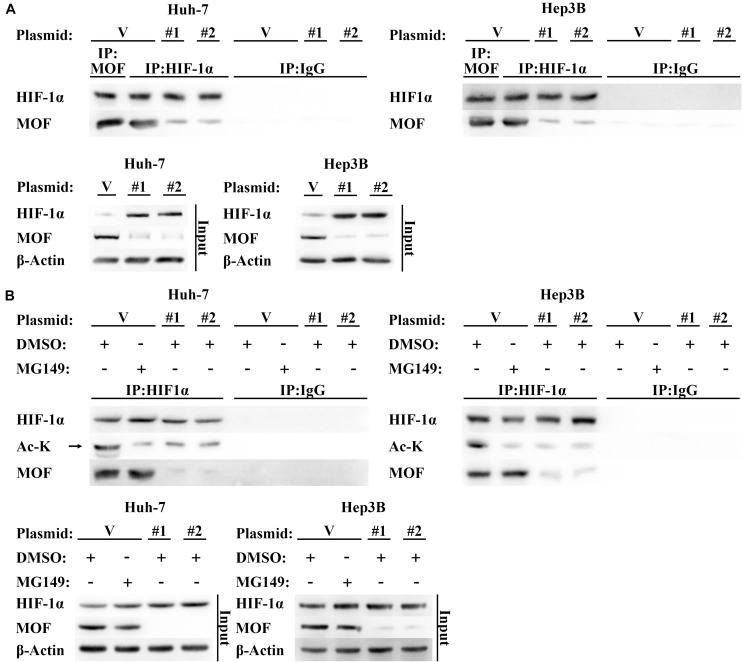
MOF interacts with HIF-1α and mediates its acetylation. **(A)** CoIP assay of MOF and HIF-1α. Mouse IgG was used as a control for MOF CoIP and rabbit IgG was used as a control for HIF-1α CoIP. Cells were pretreated with 100 μM CoCl_2_ for 24 h. **(B)** Acetylation assay to determine the effect of MOF knockdown on HIF-1α acetylation. The arrow points to the correct protein bands. Cells were treated with 100 μM CoCl_2_, together with 33 μM MG149 or equal volume of DMSO for 24 h. V, shVector; #1, shMOF #1; #2, shMOF #2.

Protein levels of HIF-1α are also known to be regulated by post transcriptional modification (Li et al., 2020). Acetylation of residues at different positions has been shown to inhibit or promote HIF-1α stability, with N-terminal acetylation leading to HIF-1α degradation (Geng et al., 2011). Due to MOF is a histone acetyltransferase that has also been shown to mediate non-histone acetylation (Li et al., 2009, 2012), we hypothesized that MOF may inhibit HIF-1α at the post-translational level via acetylation. To explore this possibility, we first performed Co-immunoprecipitation (CoIP) assays to determine whether MOF interacts with HIF-1α. The results confirmed that endogenous MOF and HIF-1α proteins had interaction between the two proteins (Figure 4A). Immunofluorescence experiments also found that MOF and HIF-1α had overlapping subcellular localizations in nucleus (Supplementary Figure 1B). Next, to evaluate the effect of this interaction on acetylation of HIF-1α, we repeated CoIP assays, with cells pretreated with MG132 (a proteasome inhibitor that prevents the degradation of HIF-1α), together with MG149 (a histone acetyltransferase inhibitor) or DMSO. We opted to use 33 μM MG149 in these experiments because the dose was within the range that inhibits MOF (47 ± 14 μM), but without the range that inhibits Tip60 (74 ± 20 μM) and other histone acetylases (>200 μM) (Ghizzoni et al., 2012; Valerio et al., 2017). The results demonstrated that 33 μM MG149 significantly decreased HIF-1α acetylation levels, and that knockdown of MOF caused a similar reduction in acetylation (Figure 4B), thus further supporting the possibility that MOF may acetylate HIF-1α.

**FIGURE 5:**
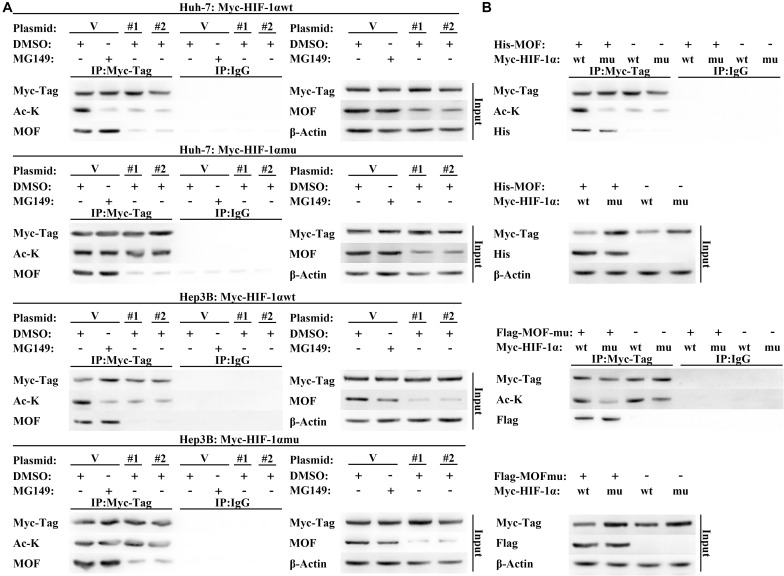
MOF regulates N-terminal acetylation of HIF-1α. **(A)** Acetylation assay to determine the effect of MOF knockdown on wild-type and mutated HIF-1α N-terminal acetylation. Cells were co-transfected with shMOF or control plasmid, and Myc-HIF-1α wild-type (Myc-HIF-1α-wt) or Myc-HIF-1α N-terminal mutation (Myc-HIF-1α-mu) vector. Mouse IgG was used as a control. **(B)** Acetylation assay to determine the effect of wild-type and mutant MOF on the acetylation of HIF-1α N-terminal lysine. Mouse IgG was used as a control. Cells were treated with 33 μM MG149 or equal volume of DMSO. V, shVector; #1, shMOF #1; #2, shMOF #2.

To verify these results, we constructed wild type (Myc-HIF-1α-wt) and N-terminal lysine mutant (Myc-HIF-1α-mu) HIF-1α expression vectors. The Myc-HIF-1α-mu vector contained five N-terminal lysine-to-arginine mutations (at K10, K11, K12, K19, and K21), which mimicked unacetylated lysine (Fujimoto et al., 2012). In contrast to the results with HIF-1α-wt, acetylation of HIF-1α-mu was not affected by MG149 treatment or MOF knockdown, suggesting that MOF-dependent acetylation of HIF-1α occurs within the N-terminal lysine residues (Figure 5A). As additional verification of these results, we used wild-type and acetyltransferase domain-mutated MOF co-expressed with wild-type and N-terminal lysine-mutated HIF-1α, which demonstrated that wild-type HIF-1α acetylation was only mediated by wild-type MOF but not by acetyltransferase domain-mutated MOF (Figure 5B). Although N-terminal mutated HIF-1α still could interact with MOF, the N-terminal acetylation level of mutated HIF-1α would not be regulated by wild-type MOF. Collectively, these results demonstrate that MOF specifically binds HIF-1α and acetylates HIF-1α at N-terminal residues.

### MOF Promotes HIF-1α Degradation

**FIGURE 6:**
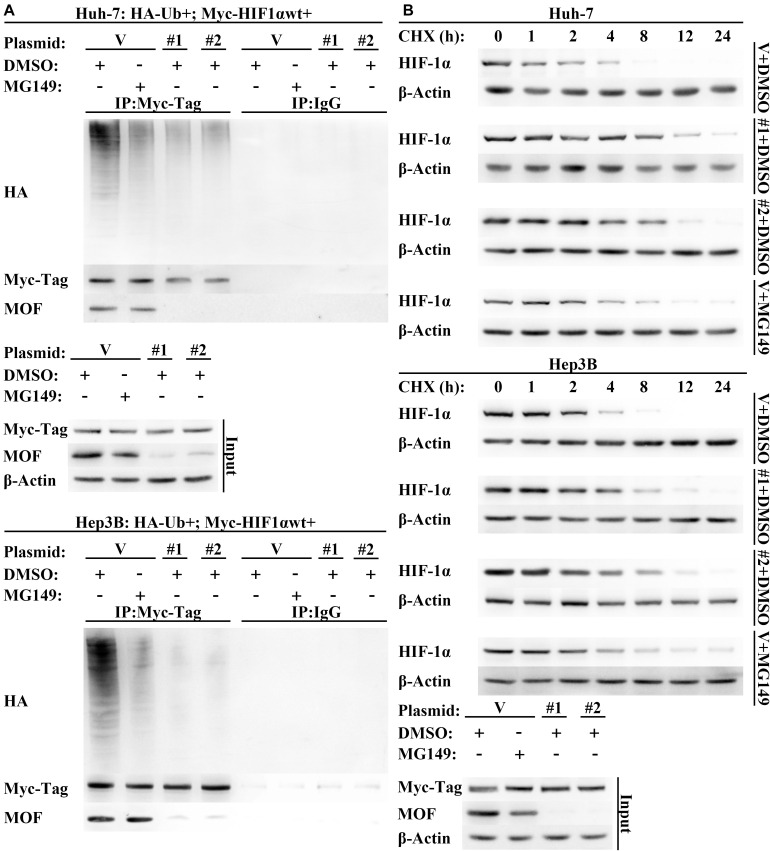
Lack of MOF function inhibits HIF-1α degradation. **(A)** Western blot assay of HIF-1α protein degradation in cells transfected with MOF or control shRNA vectors in the presence or absence of MG149. Cells were treated with 10 μg/mL CHX for indicated lengths of time. **(B)** HIF-1α polyubiquitination assay in cells transfected with MOF or control shRNA vectors. Cells were treated with 33 μM MG149 or equal volume of DMSO. V, shVector; #1, shMOF #1; #2, shMOF #2.

Though our results are consistent with the possibility that acetylation by MOF reduces HIF-1α protein expression by enhancing HIF-1α degradation, a direct demonstration of the role for MOF in destabilizing HIF-1α has not yet to be verified. Therefore, to verify that MOF modulates HIF-1α protein stability, we treated cells with the protein synthesis inhibitor cycloheximide (CHX) over a time course. The results suggested that the stability of HIF-1α was improved by knockdown of MOF, and that treatment with MG149 promoted a similar increase in HIF-1α stability (Figure 6A). Furthermore, the ubiquitination of HIF-1α was decreased in cells with lack of MOF function, either due to MG149 treatment or knockdown (Figure 6B). These results support the possibility that MOF-dependent HIF-1α N-terminal lysine acetylation promotes HIF-1α proteasomal degradation.

### The MOF-HIF-1α Axis Regulates Cell Sensitivity to CoCl_2_-Mimicked Hypoxia

**FIGURE 7:**
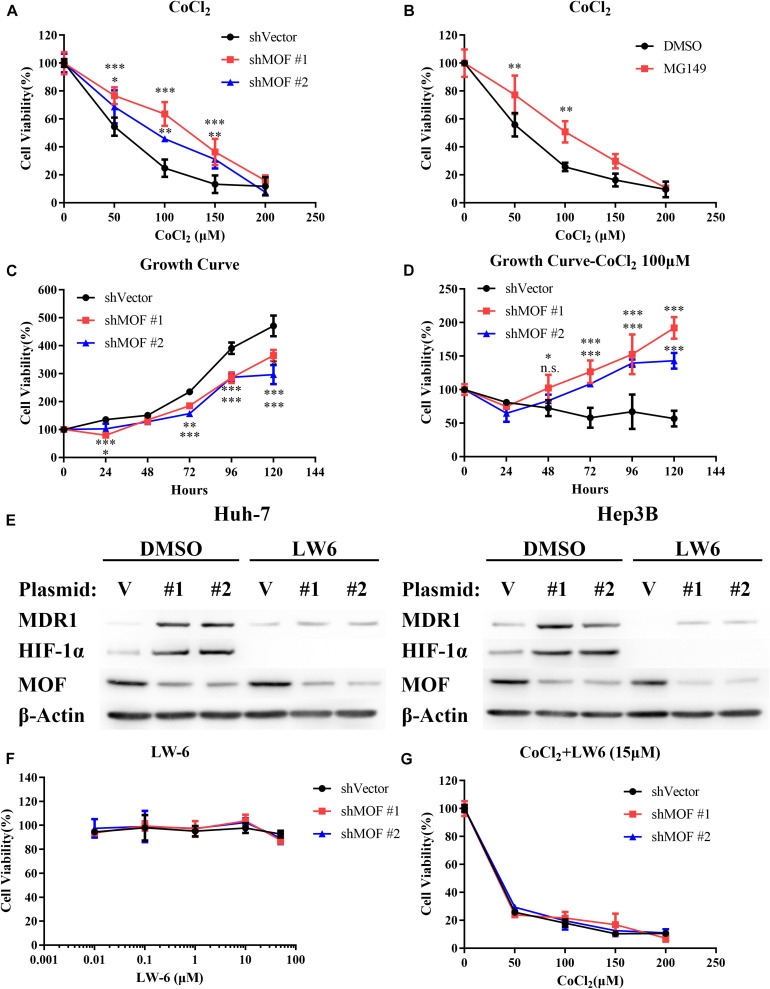
The MOF- HIF-1α axis regulates cell sensitivity to CoCl_2_-mimicked hypoxia. **(A)** Cytotoxicity assay in cells transfected with control or MOF knockdown vectors and exposed to increasing concentrations of CoCl_2_ for 72 h. **(B)** Cytotoxicity assay in the absence or presence of the acetylation inhibitor MG149 (33 μM) and with increasing concentrations of CoCl_2_ for 72 h. An equal volume of DMSO was added to control cells. **(C,D)** Cell viability assay showing relative cell growth rates at various time points in Huh-7 cells under normal conditions **(C)** or CoCl_2_-mimicked hypoxia with 100 μM CoCl_2_ **(D)**. **(E)** Western blot analysis of MOF, MDR1 and HIF-1α protein expression with 15 μM LW6 or an equal volume of DMSO treatment for 24 h. **(F)** Cytotoxicity assay after treatment with the HIF-1α inhibitor LW6 at various concentrations for 72 h. **(G)** Cytotoxicity assay after treatment with different concentration of CoCl_2_ and 15 μM LW6 for 72 h. The experiments in legends A-D and F-G were performed in Huh-7 cells. ^∗^*P* < 0.05, ^∗∗^*P* < 0.01, and ^∗∗∗^*P* < 0.001; n.s., not significant. V, shVector; #1, shMOF #1; #2, shMOF #2.

To determine whether MOF is involved in regulating HIF-1α-mediated hypoxia tolerance, we evaluated the effect of MOF knockdown on CoCl_2_-mimicked hypoxia. Notably, knockdown of MOF decreased the cell sensitivity to CoCl_2_-mimicked hypoxia by increasing cell viability under CoCl_2_ treatment in a range of doses (Figure 7A). Similar results were also obtained with MG149 (Figure 7B). To confirm that the effect of MOF knockdown on cell viability is dependent on hypoxia, we evaluated cell viability over a time course in the absence or presence of CoCl_2_. Knockdown of MOF in the absence of CoCl_2_ reduced the cell viability of Huh-7 cell line (Figure 7C); However, MOF knockdown under CoCl_2_-mimicked hypoxic conditions increased the cell viability capacity (Figure 7D). These results suggest the role for MOF in regulating cell viability is dependent on the hypoxic state.

To verify that lack of MOF reduces cell sensitivity to hypoxia by HIF-1α, we applied LW6, a novel HIF-1α inhibitor that promotes HIF-1α proteasomal degradation by activating von Hippel-Lindau (VHL) without altering HIF-1β protein level (Lee et al., 2007, 2010). After treatment with 15 μM LW6, the protein expression of HIF-1α and its downstream protein MDR1 were significantly suppressed regardless of MOF expression (Figure 7E). LW6 had no effect on cell viability at a range of concentrations (Figure 7F); however, in the presence of 15 μM LW6, MOF knockdown no longer enhanced the tolerance of cells to CoCl_2_-mimicked hypoxia (Figure 7G). These results verify that the effect of MOF in regulating hypoxia sensitivity is dependent on HIF-1α.

### MOF Knockdown Promotes TSA Resistance by Protecting HIF-1α From TSA-Induced Protein Degradation

The HDAC inhibitor TSA has been demonstrated to decrease HIF-1α protein stability and cancer cell tolerance to hypoxia (Schoepflin et al., 2016). To determine whether MOF is involved in TSA-mediated biological effects in HCC cells, we exposed Huh-7 cells to TSA after transfection with control shRNA or MOF shRNA. As expected, TSA treatment caused a significant reduction of HIF-1α; furthermore, cells transfected with MOF shRNA maintained protein levels of HIF-1α, which were accompanied by decreased Caspase 3 cleavage levels (Supplementary Figure 2A). Cells with MOF knockdown were also less sensitive to TSA treatment than cells with control shRNA (Supplementary Figure 2B). Furthermore, cells with MOF knockdown exhibited greater resistance to a combination treatment of CoCl_2_ and TSA in a range of concentrations (Supplementary Figures 2C,D). Collectively, these data show that the cytotoxic effect of TSA and its function in decreasing HIF-1α levels are dependent on MOF.

### MOF Knockdown Causes Multidrug Resistance via HIF-1α

**FIGURE 8:**
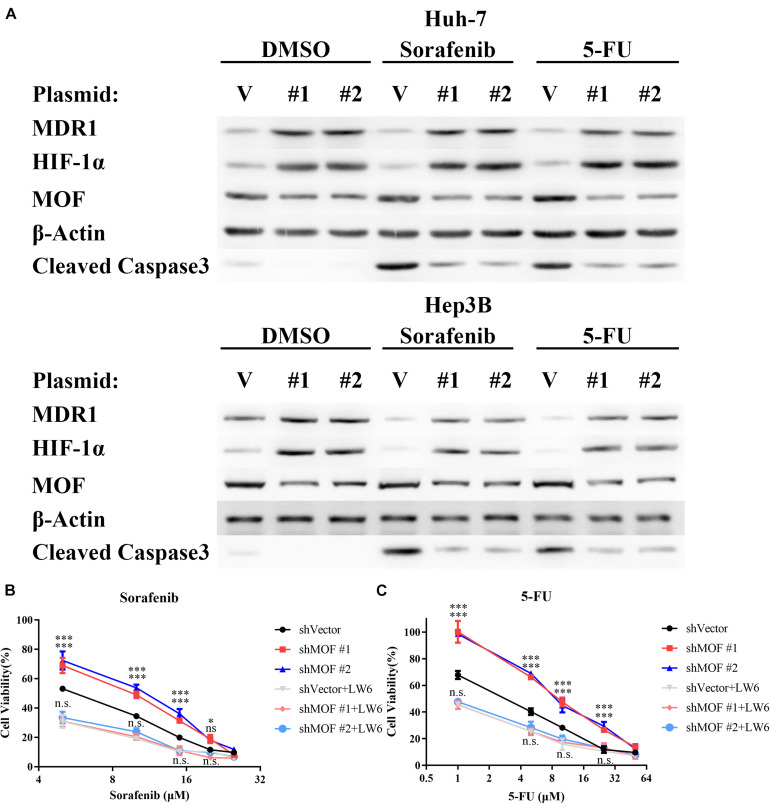
Reduction of MOF causes multidrug resistance via HIF-1α. **(A)** Western blot analysis of MOF, MDR1 and HIF-1α protein expression and Caspase 3 cleavage after treatment with DMSO vehicle, 10 μM sorafenib or 10 μM 5-FU treatment for 72 h. **(B,C)** Cytotoxicity assays for sorafenib **(B)** or 5-FU **(C)**. The experiments in legends **(B,C)** were performed on Huh-7 cells, which were treated with different concentration of the indicated drugs for 72 h. Cells with LW6 treatment were treated with 15 μM LW6. ^∗^*P* < 0.05, ^∗∗∗^*P* < 0.001 and ns, not significant as compared to shVector cells; n.s., not significant as compared to shVector + LW6 cells. V, shVector; #1, shMOF #1; #2, shMOF #2.

The RAF/MEK/ERK inhibitor sorafenib and the thymidylate synthase inhibitor 5-FU have each been approved as therapy for patients with HCC. Furthermore, HIF-1α overexpression has been demonstrated to promote HCC cell resistance to each of these drugs (Liang et al., 2013; Wu et al., 2016; Schoning et al., 2017). Therefore, we evaluated whether the reduction in MOF levels may contribute to chemoresistance in HCC. Our results confirmed that knockdown of MOF significantly elevated the protein level of HIF-1α and MDR1 during sorafenib or 5-FU treatment, and also demonstrate that MOF knockdown decreased the induction of cleaved Caspase-3 by chemotherapy (Figure 8A). Consistently, MOF knockdown increased the cell viability after treatment of sorafenib or 5-FU, whereas the addition of LW6 reversed this effect (Figures 8B,C). These results suggested that reduction of MOF induces HCC cell resistance to sorafenib and 5-FU, and that this effect was dependent on HIF-1α function.

## Discussion

Hepatocellular carcinoma is a malignant tumor with a poor prognosis, and surgery may not be an option in cases of advanced liver cancer, which is often presented with systematic metastases (Craig et al., 2020). The regulatory pathways leading to HCC progression are complex and diverse, but they also involve proliferation, hypoxia tolerance, drug resistance, tumor metastasis, and angiogenesis. Hypoxia, which is a typical feature of malignant solid tumors, is especially noted in HCC (Walsh et al., 2014; Xiong et al., 2017). Unfortunately, tumor hypoxia tolerance leads to multidrug resistance, which is directly associated with poor prognosis in advanced HCC patients. When tumor cells are under hypoxic conditions, HIF-1α is activated and regulates downstream pathways related to tumor development (Wilson et al., 2014). We have provided further understanding of this process by demonstrating that in HCC, the expression of MOF is inversely related to the expression of HIF-1α, with associated roles for MOF in tumor hypoxia tolerance.

Males absent on the first regulates tumorigenesis and tumor progression in a cell- and tissue- specific manner. In human renal cell carcinoma, primary breast carcinoma, medulloblastoma and HCC, MOF are downregulated and lack of MOF plays a critical role in tumor progression with poor outcome (Pfister et al., 2008; Wang et al., 2013; Zhang et al., 2014). However, prior to this study, the mechanism of MOF downregulation in hypoxia tolerance and chemoresistance during tumor development remains elusive. We demonstrated that in HCC cells, lack of MOF increased HIF-1α mRNA expression and HIF-1α protein stability, thus induced hypoxia tolerance and multidrug resistance. This newly demonstrated role of MOF in HIF-1α-mediated tumor hypoxia tolerance and chemoresistance may partly explain the significance of low MOF expression during tumor progression.

Our results are consistent with other studies demonstrating that the regulation of HIF-1α expression can be determined either transcriptional or post-transcriptional modification. The growth factor-activated mammalian target of rapamycin (mTOR) has been shown to increase HIF-1α protein levels through transcriptional activation of HIF-1α (Laughner et al., 2001). By contrast, posttranslational modification of HIF-1α is mainly regulated by acetylation, hydroxylation, and phosphorylation, with acetylation regulating HIF-1α protein stability either positively and negatively. N-terminal acetylation plays an important role in the degradation of HIF-1α, and deacetylation of N-terminal lysine residues by HDAC4 increases HIF-1α protein levels and enhances target gene activation (Geng et al., 2011). In contrast, p300 acetylates C-terminal K709 and K674 of HIF-1α to increase protein stability and promote transactivation of HIF-1α target genes (Lim et al., 2010; Geng et al., 2012). In this study, we demonstrated, for the first time, that lack of MOF specifically reduced N-terminal lysine acetylation of HIF-1α to promote protein degradation, and elevated mRNA expression by transcriptional regulation. Thus, in the process of hypoxia regulation, MOF regulated the protein level of HIF-1α through both transcriptional regulation and post-transcriptional modification.

We also demonstrated that knockdown of MOF in HCC cells led to decreased cell viability, but that the effect of MOF knockdown is reversed under hypoxic-mimicked conditions. Previous studies have confirmed that MOF is an important regulator of mitochondrial transcription and respiration (Chatterjee et al., 2016). MOF knockdown in HCC cells has been demonstrated to promote cell proliferation with elevated cell numbers (Zhang et al., 2014). However, because the MTT cell viability assay is based on NAD(P)H production, we believe that this does not contradict the existing results (Zeiger et al., 2011). The hypoxic conditions simulated by CoCl_2_ block cell aerobic respiration (Tripathi et al., 2019). Knockdown of MOF increases viability in a simulated hypoxic environment, which shows that knockdown of MOF promotes cell tolerance to hypoxia, thus elevating cell viability under hypoxic conditions.

Notably, MOF knockdown enhanced tolerance to TSA, a selective class I and II HDAC inhibitor, and has been used in treatment for a variety of tumors, such as cutaneous T-cell lymphoma, peripheral T-cell lymphoma (Mann et al., 2007; Sawas et al., 2015), and is currently in phase II clinical trials for HCC (Tsilimigras et al., 2018). Treatment of solid tumors with HDAC inhibitors as single agents has only shown limited therapeutic efficacy (Suraweera et al., 2018). Our results provide evidence that the cytotoxic effect of TSA and its ability to promote HIF-1α degradation are dependent on MOF. These results thus support a model in which low MOF expression in HCC cells leads to reduced HIF-1α N-terminal acetylation and thus TSA resistance. At the same time, since TSA seems to induce the ubiquitination degradation of HIF-1α through a non-VHL pathway (Qian et al., 2006; Geng et al., 2012), when we treat cells with TSA, HIF-1α in MOF-deficient cells can remain stable, whereas using the VHL pathway activator LW6, HIF-1α is degraded regardless of the expression level of MOF.

Hypoxia-inducible factor-1α regulates the transcriptional activation of the MDR1 gene to promote cell chemoresistance (Comerford et al., 2002; Ji et al., 2010), and we demonstrated that MOF indirectly regulates MDR1 expression. Sorafenib, a dual-action inhibitor that targets the RAF/MEK/ERK pathway, is used for clinical chemotherapy of HCC and significantly prolongs patient survival (Llovet et al., 2008), while 5-FU is a chemotherapy drug used to treat a variety of solid tumors, including HCC, with promising effects (Kasai et al., 2012). Our results demonstrate that dysregulation of MOF induces strong resistance to sorafenib and 5-FU in HCC cells. Therefore, the low MOF level in HCC may influence the outcome of drug treatment, which needs to be carefully considered for the future application of cancer therapy.

The present study reveals that the expression of MOF in cancer tissues is inversely related to the expression of HIF-1α. MOF inhibits HIF-1α mRNA expression through transcriptional regulation, and MOF-mediated HIF-1α N-terminal acetylation promotes HIF-1α degradation. Dysregulation of MOF leads to HCC cell tolerance to CoCl_2_-mimicked hypoxic conditions and chemotherapy by enhancing the HIF-1α accumulation. This newly identified acetylation-regulated function by MOF provides a novel target for combined therapy that may improve future HCC treatment.

## Data Availability Statement

The original contributions presented in the study are included in the article/Supplementary Material, further inquiries can be directed to the corresponding author.

## Ethics Statement

The studies involving human participants were reviewed and approved by Ethics Committee of Shandong Provincial Hospital Affiliated to Shandong University. The patients/participants provided their written informed consent to participate in this study.

## Author Contributions

MW and XZL designed the study. MW, HL, XZ, FZ, and CX performed the experiments. WZ collected the protein samples of HCC patients. XYL collected the paraffin embedded samples. MW, XZ, WZ, XYL, DL, FX, and QY analyzed the data. MW drafted the manuscript. MW, XZ, and XZL revised the manuscript. All authors have read and approved the final submitted manuscript.

## Conflict of Interest

The authors declare that the research was conducted in the absence of any commercial or financial relationships that could be construed as a potential conflict of interest.

## Publisher’s Note

All claims expressed in this article are solely those of the authors and do not necessarily represent those of their affiliated organizations, or those of the publisher, the editors and the reviewers. Any product that may be evaluated in this article, or claim that may be made by its manufacturer, is not guaranteed or endorsed by the publisher.

## Supplementary Material

Supplementary Figure 1 Subcellular localization of MOF and HIF-1α. **(A)** Nuclear and cytoplasmic protein separation experiments showed protein subcellular localization of HIF-1α with or without MOF knockdown. V, shVector; #1, shMOF #1; #2, shMOF #2. **(B)** Immunofluorescence experiments showed subcellular localizations of MOF and HIF-1α. Cells were pretreated with 100 μM CoCl_2_ for 24 h. Magnification: 1000×. Cells were treated with 100 μM CoCl_2_ or equal volume of PBS for 24 h.

Supplementary Figure 2 Lack of MOF expression protects HIF-1α from TSA-induced protein degradation and promotes TSA resistance. **(A)** Western blot analysis showing MOF and HIF-1α protein expression and Caspase 3 cleavage levels in control or MOF knockdown cells after exposure to 100 nM TSA treatment or an equal volume of DMSO for 24 h. **(B)** Cytotoxicity after exposure of control and MOF knockdown cells to TSA at a range of doses. **(C,D)** Cytotoxicity assay of TSA in Huh-7 cells under CoCl_2_-mimicked hypoxic condition. Cells were treated with different concentrations of drugs for 72 h as shown. The experiments in legends **(B–D)** were performed in Huh-7 cells. ^∗^*P* < 0.05, ^∗∗^*P* < 0.01, and ^∗∗∗^*P* < 0.001. V, shVector; #1, shMOF #1; #2, shMOF #2.

Supplementary Table 1 Primers and shRNA sequences used for vector construction in the present study.

Supplementary Table 2 Primers used in the present study for qRT-PCR.

Supplementary Table 3 MOF binding sites predicted by HOMER.
