# Supplementary figures and images for: Lack of MOF Decreases Susceptibility to Hypoxia and Promotes Multidrug Resistance in Hepatocellular Carcinoma via HIF-1α
Source: Front Cell Dev Biol. 2021 Sep 1;9:718707. doi: 10.3389/fcell.2021.718707 (PMC8440882; doi:10.3389/fcell.2021.718707)

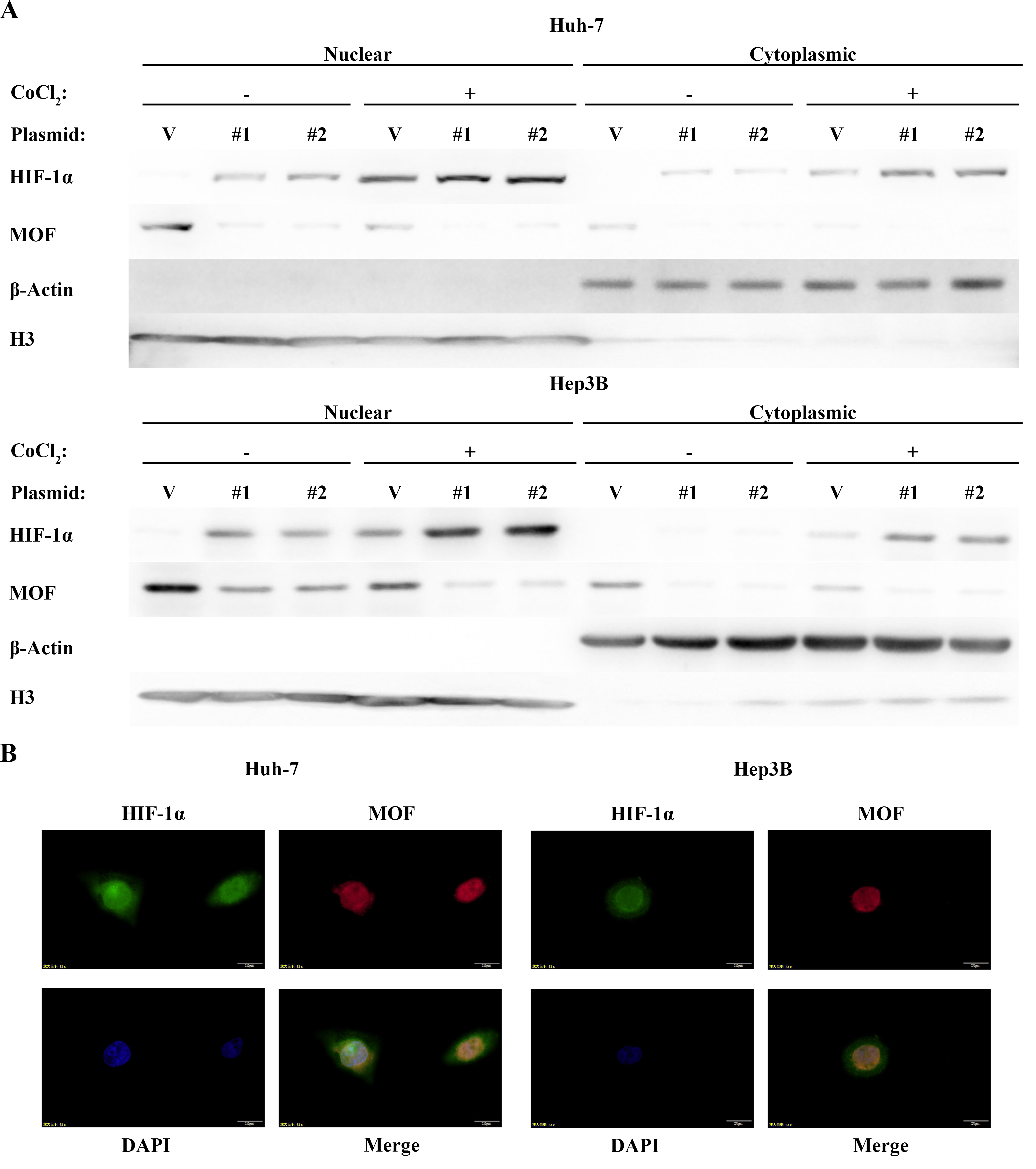

Supplement: Supplementary Figure 1 — Subcellular localization of MOF and HIF-1α. (A) Nuclear and cytoplasmic protein separation experiments showed protein subcellular localization of HIF-1α with or without MOF knockdown. V, shVector; #1, shMOF #1; #2, shMOF #2. (B) Immunofluorescence experiments showed subcellular localizations of MOF and HIF-1α. Cells were pretreated with 100 μM CoCl2 for 24 h. Magnification: 1000×. Cells were treated with 100 μM CoCl2 or equal volume of PBS for 24 h. [file Image_1.TIF]

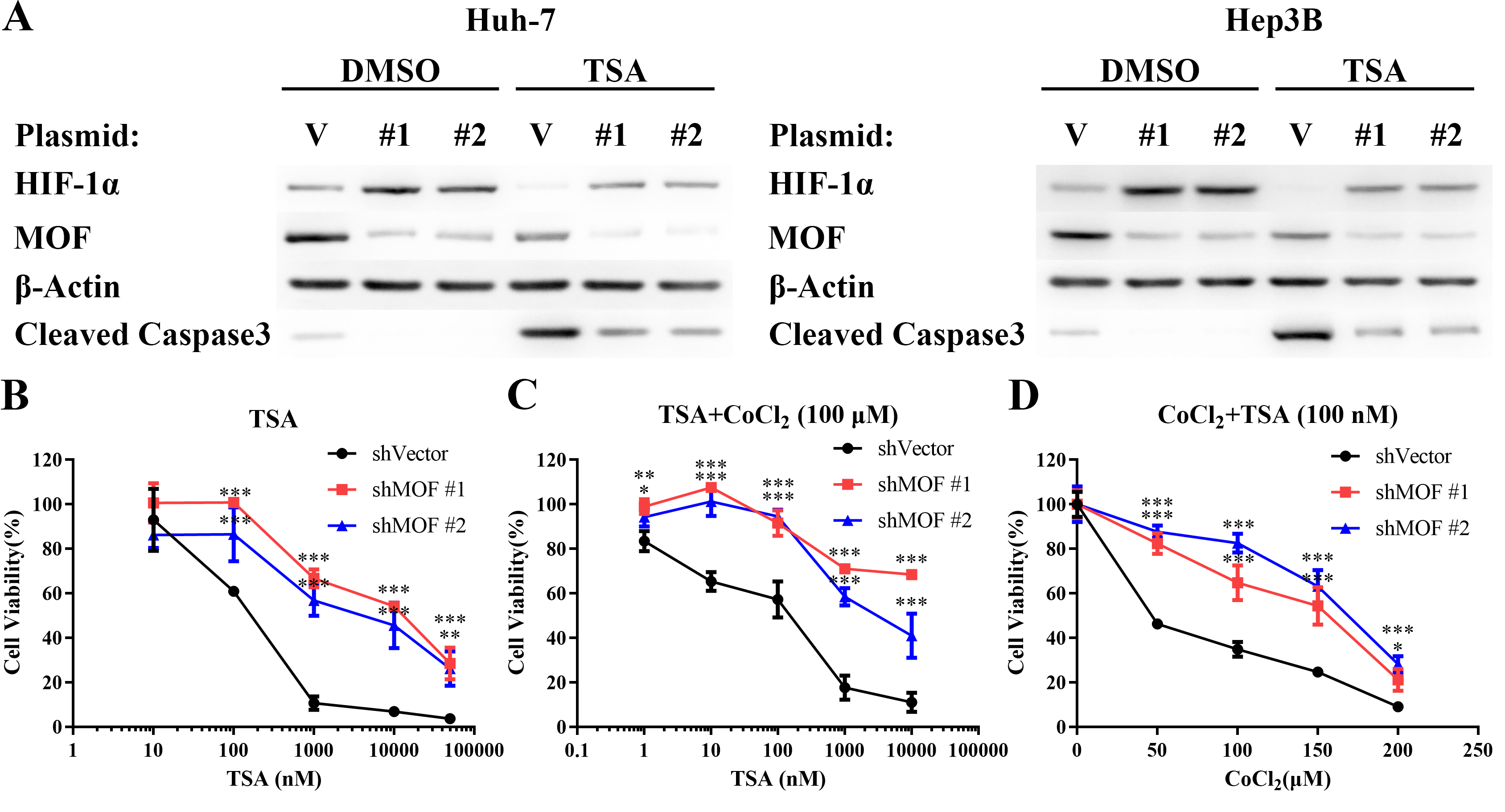

Supplement: Supplementary Figure 2 — Lack of MOF expression protects HIF-1α from TSA-induced protein degradation and promotes TSA resistance. (A) Western blot analysis showing MOF and HIF-1α protein expression and Caspase 3 cleavage levels in control or MOF knockdown cells after exposure to 100 nM TSA treatment or an equal volume of DMSO for 24 h. (B) Cytotoxicity after exposure of control and MOF knockdown cells to TSA at a range of doses. (C,D) Cytotoxicity assay of TSA in Huh-7 cells under CoCl2-mimicked hypoxic condition. Cells were treated with different concentrations of drugs for 72 h as shown. The experiments in legends (B–D) were performed in Huh-7 cells. ∗P < 0.05, ∗∗P < 0.01, and ∗∗∗P < 0.001. V, shVector; #1, shMOF #1; #2, shMOF #2. [file Image_2.TIF]
